# High-volume hemofiltration for septic acute kidney injury: a systematic review and meta-analysis

**DOI:** 10.1186/cc13184

**Published:** 2014-01-08

**Authors:** Edward Clark, Amber O Molnar, Olivier Joannes-Boyau, Patrick M Honoré, Lindsey Sikora, Sean M Bagshaw

**Affiliations:** 1 Kidney Research Centre, Ottawa Hospital Research Institute, University of Ottawa, Ottawa, Canada; 2 Division of Nephrology, The Ottawa Hospital, Ottawa, Canada; 3 Haut Leveque University Hospital of Bordeaux, University of Bordeaux 2, Pessac, France; 4 ICU Department, UniversitairZiekenhuisBrussel, VUB University, Brussels, Belgium; 5 Health Sciences Library, University of Ottawa, Ottawa, Canada; 6 Division of Critical Care Medicine, Faculty of Medicine and Dentistry, University of Alberta, 2-124E Clinical Sciences Building, 8440-122 Street, Edmonton, Alberta T6G2B7, Canada

## Abstract

**Introduction:**

High-volume hemofiltration (HVHF) is an attractive therapy for the treatment of septic acute kidney injury (AKI). Small experimental and uncontrolled studies have suggested hemodynamic and survival benefits at higher doses of HVHF than those used for the high-intensity arms of the RENAL and ATN studies. Our aim was to evaluate the effects of high-volume hemofiltration (HVHF) compared with standard-volume hemofiltration (SVHF) for septic AKI.

**Methods:**

A systematic review and meta-analysis of publications between 1966 and 2013 was performed. The review was limited to randomized-controlled trials that compared HVHF (effluent rate greater than 50 ml/kg per hour) versus SVHF in the treatment of sepsis and septic shock. The primary outcome assessed was 28-day mortality. Other outcomes assessed were recovery of kidney function, lengths of ICU and hospital stays, vasopressor dose reduction, and adverse events.

**Results:**

Four trials, including 470 total participants, were included. Pooled analysis for 28-day mortality did not show any meaningful difference between HVHF compared with SVHF (OR, 0.76; 95% CI, 0.45 to 1.29). No included studies reported statistically significant differences between groups for any of the secondary outcomes. Adverse events, including hypophosphatemia and hypokalemia, were more commonly observed in HVHF-treated patients, although reporting was inconsistent across studies.

**Conclusions:**

Insufficient evidence exists of a therapeutic benefit for routine use of HVHF for septic AKI, other than on an experimental basis. Given the logistic challenges related to patient recruitment along with an incomplete understanding of the biologic mechanisms by which HVHF may modify outcomes, further trials should focus on alternative extracorporeal therapies as an adjuvant therapy for septic AKI rather than HVHF.

## Introduction

Sepsis is a common cause of critical illness and the leading cause of death for patients admitted to the intensive care unit (ICU) [1]. It has been theorized that the removal of inflammatory mediators and/or bacterial toxins from the bloodstream could result in a beneficial downregulation of an overactive immune response that mediates end-organ damage in patients with septic shock [2-4]. As such, various forms of extracorporeal blood purification have been studied as therapeutic interventions to improve the poor outcome associated with septic shock [5,6].

The definition of what constitutes high volume in HVHF remains unclear [6,7]. Based on the results of two large randomized-controlled trials (RCTs) [8,9] and subsequent systematic reviews [10,11], the adequate dose of hemofiltration treatment for acute kidney injury (AKI) (that is, the renal dose) has been defined as an effluent rate between 25 and 30 ml/kg/hour [12]. This is consistent with HVHF being defined by an effluent rate exceeding 35 ml/kg/hour [13]. However, early experimental studies found significant hemodynamic improvements only at markedly higher effluent rates [14,15], suggesting that a cut-off of 35 ml/kg/hour may be too low [7]. Evidence supporting this notion was described in a systematic review of animal studies showing hemofiltration at “renal-dose” rates was relatively ineffective for cytokine removal [5]. Furthermore, in a *post hoc* analysis, a seminal trial found effluent rates exceeding 45 ml/kg/hour was associated with a survival benefit for a small subgroup of patients with septic AKI [16].

To address uncertainty in terms of how best to define HVHF, a consensus conference was held in Pardubice, Czech Republic, the results of which were reported in a consensus statement [17]. HVHF was defined as continuous high-volume treatment with an effluent rate of 50 to 70 ml/kg/hour (for 24 hours per day) or intermittent very high-volume treatment with an effluent rate of 100 to 120 ml/kg/hour for a 4 to 8-hour period followed by conventional renal-dose hemofiltration. This has subsequently been referred by some experts as the Pardubice consensus definition of HVHF [17].

Although the use of HVHF to treat septic AKI may hold promise, the initiation of any form of extracorporeal blood purification involves the risk of mechanical complications related to insertion of a large-bore dialysis catheter for central venous access. Other complications associated with HVHF may include hemodynamic compromise; nutrient, vitamin, and trace-metal depletion; and reduction of blood levels of antibiotics and other medications below the therapeutic range; along with technical challenges related to the time-dependent loss of hemofilter efficiency and bedside nursing workload [6,17].

Given that any benefit might be seen at higher doses of hemofiltration, and that the previously mentioned risks of harm might also be increased, we sought to perform a systematic review of randomized controlled trials that assessed the use of hemofiltration for sepsis at doses greater than the renal-dose in accordance with the Pardubice definition of HVHF.

## Methods

This study is reported according to Preferred Reporting Items for Systematic Reviews and Meta-Analyses (PRISMA) statement recommendations [18]. See Additional file 1 for a copy of the PRISMA checklist.

### Search methods for identification of studies

The following databases were electronically searched: Cochrane Database of Systematic Reviews, DARE (Database of Abstracts of Reviews of Effects), Cochrane Central Register of Controlled Trials (CENTRAL), Medline and Medline in Process (via OVID), and Embase (via OVID). A search strategy was developed to define key words for all searches (see Additional file 2 for Medline search). Abstracts from recent major conferences of leading nephrology and critical care organizations (American Society of Nephrology, International Society of Nephrology, American Thoracic Society, European Society of Critical Care Medicine, Society of Critical Care Medicine) from the past 5 years were also searched for relevant abstracts. The search included articles published on or before July 5, 2013. There were no language restrictions.

### Selection of studies

All citations were initially screened by two authors (EC and AM) to select articles for full-text review. Any disagreement regarding the final selection of studies for inclusion was resolved by consensus with a third investigator (SMB). Studies were selected according to the following parameters:

### Inclusion criteria

•RCT design

•Patients admitted to ICU

•Adults age ≥18 years

•Diagnosis of sepsis/septic shock

•Report specifically on outcomes of comparator/control group

•Treatment with HVHF defined according to an effluent rate of ≥50 ml/kg/hour, continuously, or intermittent very high-volume treatment with an effluent rate of 100 to120 ml/kg/hour for a 4- to 8-hour period followed by conventional renal-dose continuous renal-replacement therapy (CRRT).

•Reporting of the primary outcome (mortality) for any time point.

### Exclusion criteria

•Observational studies, quasi-randomized or crossover studies, case reports, case-series, use of historical controls, review articles

•Neonatal/pediatric population

•Post-cardiac surgery population

### Data extraction

Data were independently extracted from full-text articles by two authors (EC and AM). After extraction, data were reviewed and compared by EC, with disagreements solved by consensus.

### Definition of end points

The primary end point was mortality (28-day and at any time). Secondary outcomes considered were recovery of kidney function (that is, dialysis independence), hemodynamic profile (that is, reduced vasopressor requirements or improved blood pressure), proportion with shock reversal and/or time to shock reversal, organ-failure burden (for example, sequential organ failure assessment (SOFA), acute physiology and chronic health evaluation (APACHE) II scores), and lengths of stay (ICU, hospital). Adverse events that may be therapy related were also considered as secondary end points. These included electrolyte, nutrient, vitamin, and trace-metal depletion, and reduction of blood levels of antibiotics and other medications below the therapeutic range.

### Assessment of methodologic quality

All included studies were assessed by using Jadad scoring (the Oxford quality scoring system) for clinical trials [19], which encompasses the reporting of randomization technique, presence and appropriateness of blinding, and description of dropouts and withdrawals. In addition, we considered whether analysis was conducted according to the “intention-to-treat”, if an *a priori* defined protocolization of interventions and *a priori* defined primary and secondary end points.

### Statistical analysis

Data analysis was performed by using Review Manager, version 5.0 (RevMan; The Nordic Cochrane Centre, The Cochrane Collaboration 2008, Copenhagen, Denmark). For the outcome of 28-day mortality, data from included studies was combined by using a random-effects model expressed as an odds ratio with a 95% confidence interval. The level of statistical significance was set at *P* < 0.05. This analysis was also conducted by using a fixed-effects model to evaluate for robustness and susceptibility to outliers. Statistical heterogeneity was quantified for the pooled result by using the τ^2^, χ^2^, or I^2^ statistics. No statistical evaluation for publication bias was performed.

## Results

### Literature search

**Figure 1:**
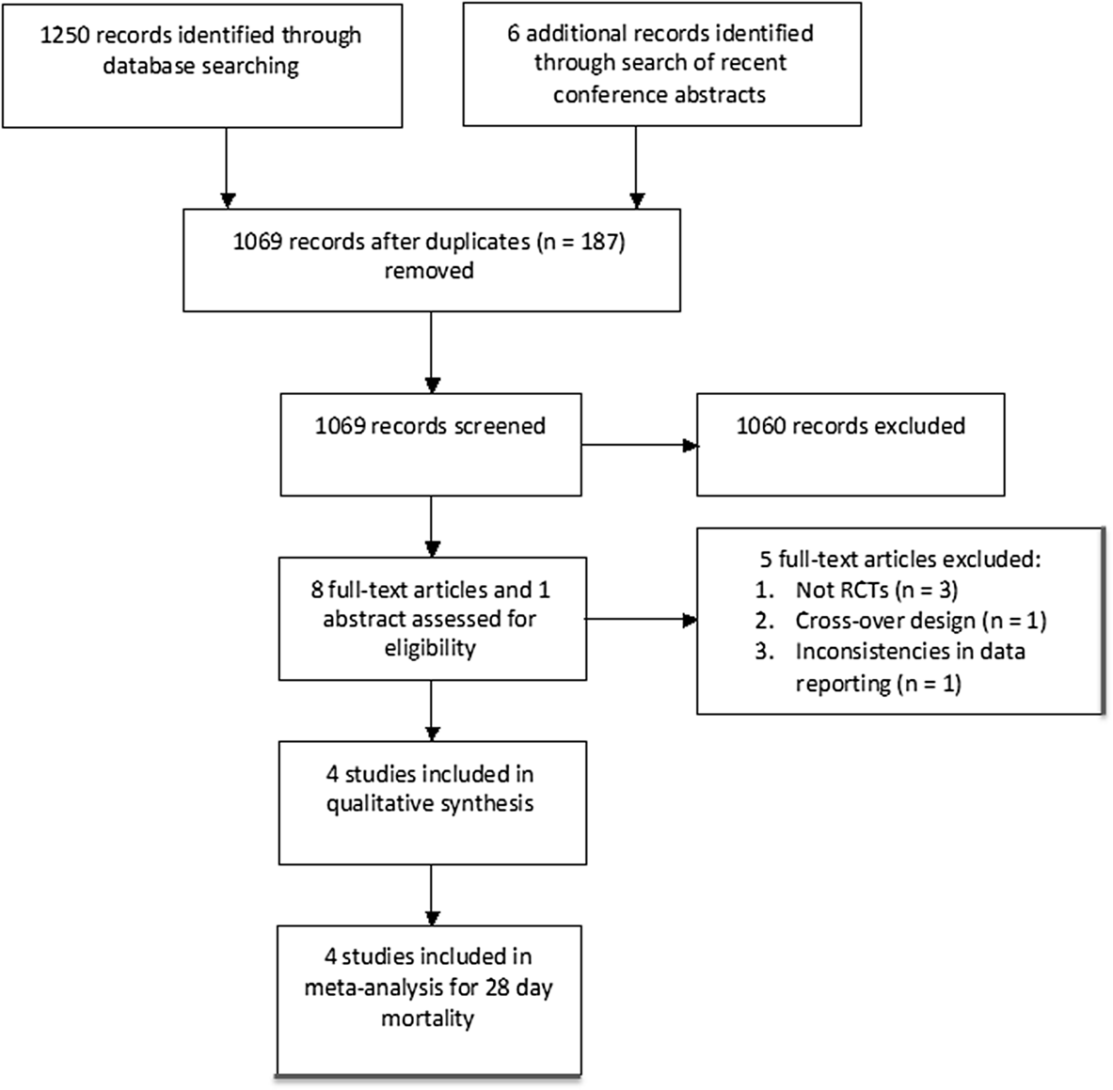
**Flow diagram of process for identification of studies.** Template Modified from: Moher D, Liberati A, Tetzlaff J, Altman DG, The PRISMA Group (2009). *P*referred *R*eporting *I*tems for *S*ystematic Reviews and *M*eta-*A*nalyses: The PRISMA Statement. PLoS Med 2009, **6**: e1000097. doi:10.1371/journal.pmed1000097 [18].

Our search of Medline yielded 327 citations; Embase, 545 citations; and Cochrane and DARE databases, 12 citations. The search of conference abstracts identified five abstracts that were found to be “duplicate” abstracts related to full-text publications. After removing duplicates (*n* = 187), the search strategy identified 1,068 citations. Figure 1 is a flow diagram detailing the process by which studies were identified.

After initial screening, eight studies were obtained for full-text review. Of those, four fulfilled eligibility and were included. Four studies were excluded for the following reasons: one was a cross-over study [20], three were not RCTs [21,22], and, as previously noted by Borthwick *et al*. [23], for one study [24], the mortality rate quoted was for an unspecified time point, and the number of deaths did not correspond to reported mortality rates.

### Study characteristics and quality

The characteristics and quality assessment of included studies are detailed in Table 1. The studies by Boussekey *et al*. [25], Joannes-Boyau *et al*. [26], and Sanchez *et al*. [27] assessed HVHF in patients with septic shock and AKI, whereas the study by Zhang *et al.*[28] enrolled patients with severe sepsis and AKI. No included study was blinded. Three studies were analyzed according to intention-to-treat, featured *a priori*-defined protocolization of interventions and *a priori*-defined primary and secondary end points [25,26,28]; whereas these details were not described for Sanchez *et al*. [27]. The study by Joannes-Boyau *et al*. was the only multicenter study and used block computer randomization [26].

**Table 1:** Summary of included studies

**Study**	**Journal**	**Location**	**Setting**	**Jadad scale**^**a**^	**Primary end point**	**Follow-up**
**Boussekey (2008)**[25]	*Intensive Care Medicine*	France	Single-center ICU	3	75% decrease in vasopressor dose after 24 hours	28 days
**Sanchez (2010)**[27]	*Intensive Care Medicine (abstract)*	Spain	Single-center ICU	1	All-cause mortality at 28 days	-
**Zhang (2012)**[28]	*Nephrology Dialysis Transplantation*	China	Single-center ICU	2^b^	All-cause mortality at 28 days^c^	90 days
**Joannes-Boyau (2013)**[26]	*Intensive Care Medicine*	France, Belgium, the Netherlands	18 ICUs	3	All-cause mortality at 28 days	90 days

^**a**^Jadad scale for quality appraisal (total possible score, 5) [19].

^b^No discussion of dropouts or withdrawals, but none occurred.

^c^Stated primary end point “death from any cause within 28, 60, and 90 days after randomization”.

Table 2 details the baseline characteristics of study participants. Details regarding HVHF and SVHF treatment are reported in Table 3. A substantial difference in the time from ICU admission to enrollment was observed between studies.

**Table 2:** Baseline patient characteristics of included studies

**Study**	***n***	**Randomized to HVHF: *n *** **(%)**	**Mean age (years)**		**Male gender: (*n *, %)**		**Mean sCr (μ *M *)**		**Mean APACHE II score**		**Mean SAPS II score**	
			**HVHF**	**Control**	**HVHF**	**Control**	**HVHF**	**Control**	**HVHF**	**Control**	**HVHF**	**Control**
**Boussekey (2008)**[25]	19^a^	9 (47)	68	72.5	7 (78)	8 (80)	205	191	31	33.5	68	67
**Sanchez (2010)**[27]	30	15 (50)	59 (13)		21 (70)		-	-	-	-	-	-
**Zhang (2012)**[28]	280	141 (50)	57	60	83 (59)	89 (64)	248	263	22	23	NR	NR
**Joannes-Boyau (2013)**[26]	137^b^	66 (48)	68	70	68 (45)	54 (38)	227	210	NR	NR	68	64

*sCr,* serum creatinine.

^a^Number of patients included for analysis. Number randomized was 20.

^b^Number of patients included for analysis. Number randomized was 140.

NR, not reported. NB, For all studies, no statistically significant differences were found in baseline patient characteristics (as reported earlier) between HVHF and control groups.

**Table 3:** Details of high-volume and standard-volume hemofiltration for included studies

**Study**	**Modality**	**Prescribed effluent rate (ml/kg/hr)**		**Delivered effluent rate (ml/kg/hr)**		**Days in ICU before Enrolment**		**Duration of HF (days)**	
		**HVHF**	**Control HF**	**HVHF**	**Control HF**	**HVHF**	**Control HF**	**HVHF**	**Control HF**
**Boussekey (2008)**[25]	CVVH	65	35	62	32	Not stated^a^	Not stated^a^	7	6
**Sanchez (2010)**[27]	CVVH	55	35	-	-	-	-	5.7	6.4
**Zhang (2012)**[28]	CVVH	85	50	87.54	49.99	5.4	6.2	9.38	8.88
**Joannes-Boyau (2013)**[26]	CVVH	70	35	65.6	33.2	2.4	1.9	6^b^	7^b^

CVVH*,* continuous veno-venous hemofiltration. ^a^Boussekey *et al.*[25] reported “time from shock to hemofiltration”: 21 hours for HVHF; 15.5 hours for SVHF. ^b^This was estimated according to the outcome ”RRT-free days at day 90”.

### Mortality outcomes

**Figure 2:**
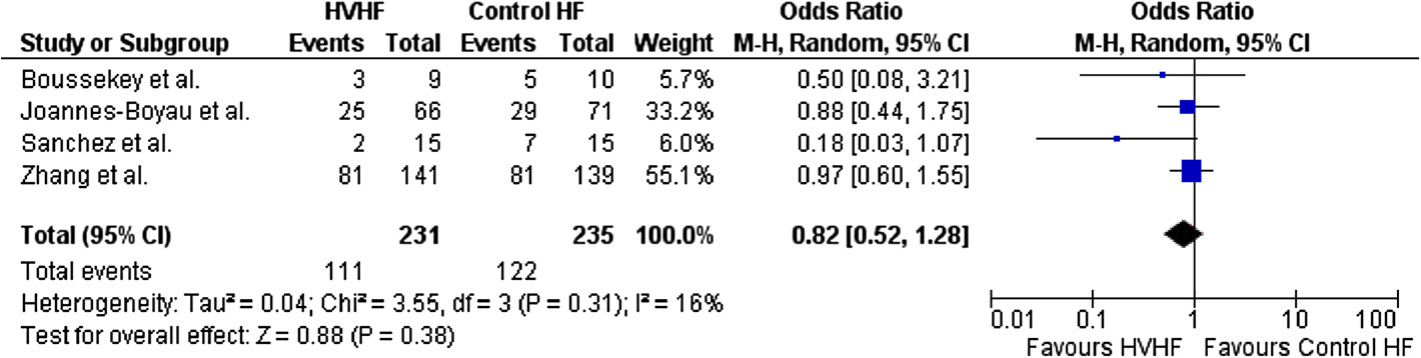
Forest plot for odds of 28-day mortality.

No study showed a statistically significant reduction in mortality for HVHF compared with SVHF. The study by Boussekey *et al*. [25] was not designed for mortality as a primary outcome; however, reported 28-day mortality as a secondary outcome. The other three studies specified 28-day mortality as a primary outcome [26-28]. For the four included studies, the pooled odds ratio (95% CI) for 28-day mortality for HVHF compared with SVHF was 0.76 (95% CI, 0.45 to 1.29; *P* = 0.31) (Figure 2). No significant heterogeneity was observed across studies for 28-day mortality (τ^2^ = 0.08; χ^2^ = 4.19 (*P* = 0.24); І^2^ = 28.4%).

The studies by Zhang *et al.*[26] and Joannes-Boyau *et al.*[28] also reported 60- and 90-day mortality [26,28]. In both studies, HVHF had no discernible impact on mortality at 60 or 90 days compared with SVHF.

### Secondary outcomes

#### Vasopressor dose reduction

Boussekey *et al*. [25] showed more patients who received HVHF (eight of nine) had a 75% reduction in norepinephrine dose within 24 hours compared with those who received SVHF (four of 10) (relative risk (RR) 2.22 (95% CI, 1.01 to 4.51) [25]. The study by Zhang *et al*. [28] showed no significant differences in norepinephrine doses before and after treatment with HVHF or SVHF [28]. Joannes-Boyau *et al*. [26] found no effect of HVHF on vasopressor dependency with an RR (for the Log Vasopressor Dependency Index) of 1.005 (95% CI, 0.99 to 1.02) [26]. The heterogeneous measures used to determine vasopressor reduction across studies did not allow pooled analysis.

#### Recovery of kidney function

Boussekey *et al*. [25] reported all surviving patients had recovery of kidney function by ICU discharge. The studies by Zhang *et al*. [25] and Joannes-Boyau *et al*. [28] reported no significant differences in the proportion of survivors with dialysis dependence at 90 days. Zhang *et al*. [28] reported no significant difference in dialysis dependence among survivors at 90 days (HVHF, 7% (four of 57) versus SVHF 10% (five of 51)) [28]. For the study by Joannes-Boyau *et al*. (26), no patient of 29 in the HVHF group and 3% (one of 35) in the SVHF group were dialysis dependent at 90 days.

#### Lengths of stay

No study demonstrated significant differences or important trends in ICU or hospital lengths of stay between HVHF and SVHF groups, respectively.

#### Adverse events

Two studies did not describe adverse events [25,27]. In the study by Zhang *et al*. [28], adverse events were not specifically described; however, it was noted that three patients in the HVHF group and one in the SVHF had hypothermia, defined as a core temperature <34°C while receiving CRRT [28]. Associated with this, core temperature was significantly lower in the HVHF compared with SVHF group (37.2°C versus 37.9°C; *P* < 0.001). The authors also described that hypophosphatemia occurred more commonly among HVHF-treated patients (65%) compared with SVHF (54%); however, hypophosphatemia was not specifically defined [28].

The study by Joannes-Boyau *et al*. [26] reported three adverse events (one acute embolic stroke, one myocardial infarction, and one episode of major postoperative bleeding). One major adverse event occurred in the HVHF and two in the SVHF group, all independently adjudicated to be unrelated to the study intervention. A trend toward more hypokalemia in the HVHF-treated patients was found (30% versus 20%; *P* = 0.1), whereas more episodes of hypophosphatemia occurred in HVHF-treated compared with SVHF-treated patients (HVHF, 97 events, 88% (*n* = 32) versus SVHF: 43 events, 38% (*n* = 34); *P* < 0.01).

Neither Zhang *et al*. or Boussekey *et al*. described antimicrobial clearance, although Zhang *et al*. indicated that the dosing of antibiotics was “adjusted according to clearance of HVHF during the intervention period” [25,28]. The study by Joannes-Boyau *et al*. reported administering standard, non-AKI doses of antibiotics in both arms and that the mean elimination half-life of antimicrobials in the HVHF group was significantly shorter than that in the SVHF group (1.3 to 28.5 hours versus 1.5 to 33.9 hours) [26].

## Discussion

A systematic search of the literature for randomized controlled trials evaluating HVHF, according to the Pardubice definition [29], compared with SVHF as adjuvant therapy for sepsis and septic AKI, found only four eligible studies for analysis. The quality of each study included in our review was reasonable, considering the inherent challenges for blinding an extracorporeal therapy. Our review found that HVHF, compared with SVHF, had no significant impact on short-term mortality, kidney recovery, improvement in hemodynamic profile, or reduction in ICU or hospital length-of-stay. In a pooled analysis evaluating 28-day mortality, HVHF was associated with a small but nonsignificant trend toward benefit compared with SVHF.

The use of an extracorporeal therapy to augment inflammatory mediator clearance is conceptually appealing; however, numerous plausible explanations exist for the apparent lack of efficacy and potential for harm with HVHF. First, sepsis and septic AKI are characterized by an excess production of both pro- and antiinflammatory mediators [4]. HVHF using conventional hemofilters may simply be ineffective at providing either sufficient or sustained mediator clearance to show measurable benefit [5]. Second, given the critical importance of timely [30] and appropriate [31] antimicrobial therapy for the treatment of sepsis, any excess clearance of antimicrobials with HVHF may contribute to subtherapeutic plasma concentrations and predispose to treatment failure or risk of worse outcome [26]. Finally, HVHF is associated with higher rates of electrolyte abnormalities (for example, hypophosphatemia, hypokalemia) and excess micronutrient depletion compared with SVHF, that may further confound the association between any therapeutic benefit and outcome [8,26]. Recent data suggested that the development of hypophosphatemia during CRRT may portend increased risk for less-favorable outcomes [32,33]. Finally, although not specifically discussed in any included study, the bedside application of HVHF is far more resource intensive for nurses and may add considerable expense beyond conventional RRT (that is, because of replacement fluid costs). As such, given the evidence of a lack of efficacy found in the available higher-quality trials included in our review, coupled with the concerns for potential adverse effects and added expense, the utility of HVHF should be questioned as an adjuvant therapy for critically ill patients with sepsis and septic AKI.

Our review has several important limitations. First, using the Pardubice definition of HVHF may not have captured all RCTs evaluating HVHF compared with SVHF. We also considered that both the ATN and RENAL trials [8,9] found no evidence of benefit for higher-intensity RRT (defined as effluent rates of 35 and 40 ml/kg/hr) for the subgroup with sepsis (723 patients (48%) with severe sepsis in the RENAL trial; 708 patients [63%] with sepsis in the ATN trial). However, we believed a focused evaluation of HVHF characterized by effluent rates exceeding the intervention arms of these trials was necessary, based on data suggesting that HVHF may be commonly applied in clinical practice [34]. Second, studies fulfilling eligibility for our review were heterogeneous. For example, the control group in the study by Zhang *et al*. [28] prescribed a relatively high intensity of HF (prescribed at 50 ml/kg/hr) that approached the definition of HVHF, according to the Pardubice definition. As such, the authors refer to the higher dose as being extra-high-volume hemofiltration (EHVHF) and to the control arm of HVHF. After discussion, we believed this study fulfilled eligibility based on the rationale that the prescribed dose for this study was only marginally lower than the high-intensity arms of the RENAL and ATN trials, which prescribed a lower effluent dose and did not show improved outcome [8,9,28]. Similarly, the trials included in our analysis showed substantial variation in the timing of initiation of the intervention. Whereas it is not possible to draw definitive conclusions regarding the impact of variation on the initiation and outcomes in these studies, timing of therapy may be an important confounder of the association between RRT and outcome, in particular, renal recovery [35].

Although we focused on RCTs for this analysis, it should be noted that recent reviews of this subject have taken a broader approach. As detailed in a recent review [6], both retrospective [36] and prospective cohort studies [37-39] have suggested improved 28-day survival compared with historical controls or survival calculated from illness-severity scores. Using a definition of HVHF of ultrafiltration rate ≥35 ml/kg/hr [16] and including studies with a quasi-randomized design, a recent Cochrane Review included only three studies (64 participants) [23]. This review included the studies by Boussekey *et al*. and Ghani *et al*. (in which problems were identified with the reporting of mortality data) [24,25]. Further, the small crossover trial reported by Cole *et al*. [20] was included in their analysis, despite no description of mortality outcomes. No pooled analysis was performed, and the authors concluded that the evidence base was “very weak” in “support [of] the use of HVHF in critically ill patients with severe sepsis/septic shock” [23].

Our review also included the study by Boussekey *et al*.; however, it was strengthened by the inclusion of the three recently reported trials [26-28]. We similarly conclude that the evidence base to support routine adjuvant therapy in septic AKI with HVHF is weak. Unlike previous reviews on this issue; we further conclude that HVHF is unlikely to be sufficiently beneficial to justify a large, resource-intensive randomized controlled trial [6,23]. This may be more relevant, given the recruitment challenges with an intervention of this nature, as described in the IVOIRE trial [26]. It could be argued that the benefits of HVHF compared with SVHF observed in uncontrolled trials, [36-39] in animal studies [14,40,41] and on surrogate outcomes [20] provide a basis to merit further RCTs despite the difficulties in patient recruitment. Given the dangers of relying on surrogate end points in making conclusions about clinical efficacy [42] and the recent history of the failure of surrogate end points in critical care in particular [43-46], we believe that further trials of HVHF compared with SVHF for septic AKI are not indicated in light of the RCT evidence that we have reviewed herein.

In our opinion, the complexity of the disease process under study (human sepsis with acute kidney injury) and the incompletely understood nature of the proposed therapeutic effect of HVHF [4,47] make it a field of study particularly vulnerable to reliance on surrogate end points that may subsequently be proven to be invalid [42]. A recent *post hoc* analysis of a nested cohort of 115 patients from the RENAL study demonstrated that those in the higher-intensity arm (40 ml/kg per hour) had improved blood pressure and decreased vasopressor requirements in the absence of other changes that might explain the effect, such as acid–base or temperature differences compared with the control arm [48]. We agree with the authors’ conclusion that investigation of the mechanism underpinning the hemodynamic improvement observed might provide insight into future therapeutic interventions [48].

Finally, we acknowledge that our review is limited by a paucity of high-quality evidence and only included four randomized trials, three of which were equivocal. However, a statistical note describing the trial by Joannes-Boyau *et al*. [49] further implied their findings were likely definitive, despite only achieving 30% of planned recruitment, given no evidence of any difference in outcome through 90 days and the low probability that this finding would change if more participants had been enrolled.

We suggest that a greater understanding of the biologic mechanisms whereby HVHF may exert a therapeutic benefit or alternatively concomitantly contribute harm should be undertaken before conducting further large-scale RCTs to assess its utility as an adjuvant therapy in septic AKI. In the meantime, studies evaluating the efficacy of various specialized extracorporeal techniques (for example, high-cutoff hemofilters, hemoadsorption) as adjuvant therapies in sepsis may prove more promising [29].

## Conclusions

A systematic review of the literature included four RCTs that evaluated the use of HVHF compared with SVHF as an adjuvant therapy for sepsis. Our review is strengthened by the addition of two large recently published RCTs, including a total of 470 patients for analysis. Based on our review, insufficient evidence exists to suggest a therapeutic benefit for routine use of HVHF in sepsis other than on an experimental basis. Importantly, no study included in our review specifically compared HVHF with standard “renal-dose” RRT, based on current best evidence (that is, the control arms of the RENAL and ATN trials [8,9]). As such, no specific recommendation regarding the use of HVHF in sepsis can be supported with high-quality evidence. Moreover, considering the logistical challenges related to patient recruitment coupled with the gaps in our understanding of the biologic mechanisms of how HVHF may improve outcome, further studies should focus on clarifying these mechanisms, or future studies should focus on alternative extracorporeal therapies as an adjuvant therapy for septic AKI rather than HVHF [5].

## Key messages

•Our systematic review recovered only four reasonable-quality trials for inclusion comparing HVHF and SVHF.

•HVHF as adjuvant therapy for sepsis and septic AKI, compared with SVHF, showed no significant impact on short-term mortality or kidney recovery.

•HVHF as adjuvant therapy for sepsis and septic AKI, compared with SVHF, was also not associated with sustained improvement in hemodynamic profile or reduction in ICU or hospital length-of-stay.

•Based on the findings of our review, no specific recommendation regarding the routine use of HVHF in sepsis and septic AKI can be supported.

•Future studies should focus on alternative extracorporeal therapies as adjuvant therapies for sepsis and septic AKI rather than HVHF.

## Abbreviations

AKI: acute kidney injury; APACHE: Acute Physiology and Chronic Health Evaluation; CI: confidence interval; CRRT: continuous renal-replacement therapy; CVVH: continuous veno-venous hemofiltration; EHVHF: extra-high-volume hemofiltration; HVHF: high-volume hemofiltration; ICU: intensive care unit; PRISMA: Preferred Reporting Items for Systematic Reviews and Meta-Analyses; RCT: randomized controlled trial; RRT: renal replacement therapy; SOFA: Sequential Organ Failure Assessment; SVHF: standard-volume hemofiltration.

## Competing interests

This study was not funded. Drs. Bagshaw, Joannes-Boyau, and Honoré have consulted for Gambro.

## Authors’ contributions

EC designed the study, conducted the search, extracted data, performed statistical analysis, and drafted the manuscript. AM conducted the search, extracted the data, and revised the manuscript. OJB interpreted data and revised the manuscript. PH interpreted data and revised the manuscript. LS conducted the literature search, extracted data, and revised the manuscript. SMB conceived the study, participated in the design, search, data extraction, statistical analysis and revised the manuscript. All authors read and approved the final manuscript.

## Supplementary Material

Additional file 1 PRISMA checklist. Click here for file

Additional file 2 Medline Search Strategy. Click here for file
