# Supplementary material for: High-volume hemofiltration for septic acute kidney injury: a systematic review and meta-analysis
Source: Crit Care. 2014 Jan 8;18(1):R7. doi: 10.1186/cc13184 (PMC4057068; doi:10.1186/cc13184)
Supplement: Additional file 2 — Medline Search Strategy. [file cc13184-S2.doc]

**Additional file 2:** Medline Search Strategy

1. Intensive Care Units/

2. Burn Units/

3. Respiratory Care Units/

4. (intensive adj care adj unit*).tw.

5. [icu.tw](http://icu.tw/).

6. 1 or 2 or 3 or 4 or 5

7. exp Sepsis/

8. (septic adj shock).tw.

9. pyohemia*.tw.

10. septicemia*.tw.

11. (blood adj poisoning*).tw.

12. pyemia*.tw.

13. [sepsis.tw](http://sepsis.tw/).

14. (distributive adj shock).tw.

15. 7 or 8 or 9 or 10 or 11 or 12 or 13 or 14

16. (high adj volume adj hemofiltration).tw.

17. (high adj dose adj h?mofiltration).tw.

18. exp Hemofiltration/

19. h?mofiltration.tw.

20. h?modialysis.tw.

21. h?modialfiltration.tw.

22. [hvhf.tw](http://hvhf.tw/).

23. exp Renal Dialysis/

24. Dialysis/

25. 16 or 17 or 18 or 19 or 20 or 21 or 22 or 23 or 24

26. 6 and 15 and 25
